# Supplementary material for: Unappreciated subcontinental admixture in Europeans and European Americans and implications for genetic epidemiology studies
Source: Nat Commun. 2023 Nov 7;14:6802. doi: 10.1038/s41467-023-42491-0 (PMC10630423; doi:10.1038/s41467-023-42491-0)
Supplement: Supplementary file 1 — Supplementary Information [file 41467_2023_42491_MOESM1_ESM.pdf]

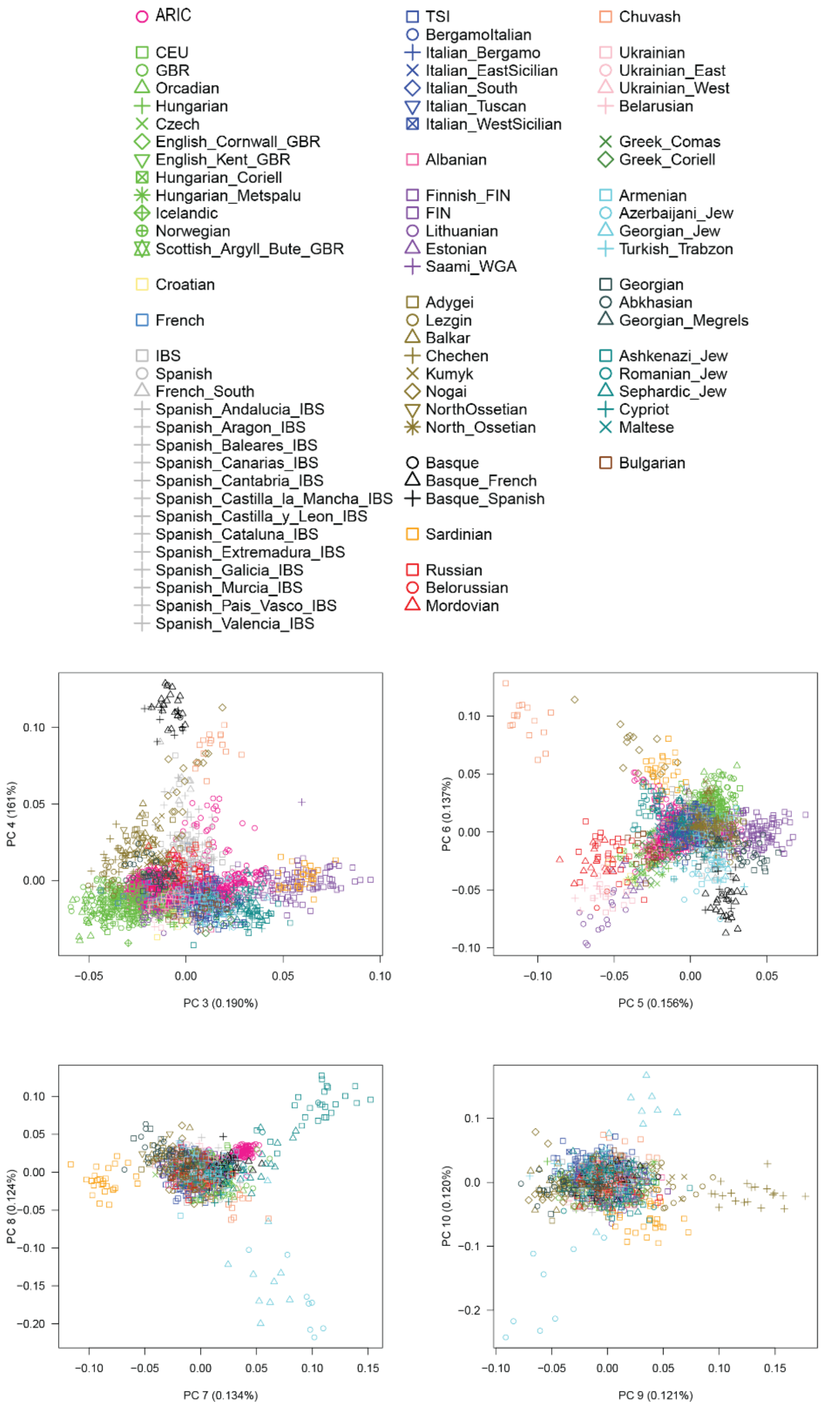

Fig. S1. Principal component analysis (principal components 3 through 10) of the European reference panel. European Americans (ARIC cohort) were projected onto the European reference panel.

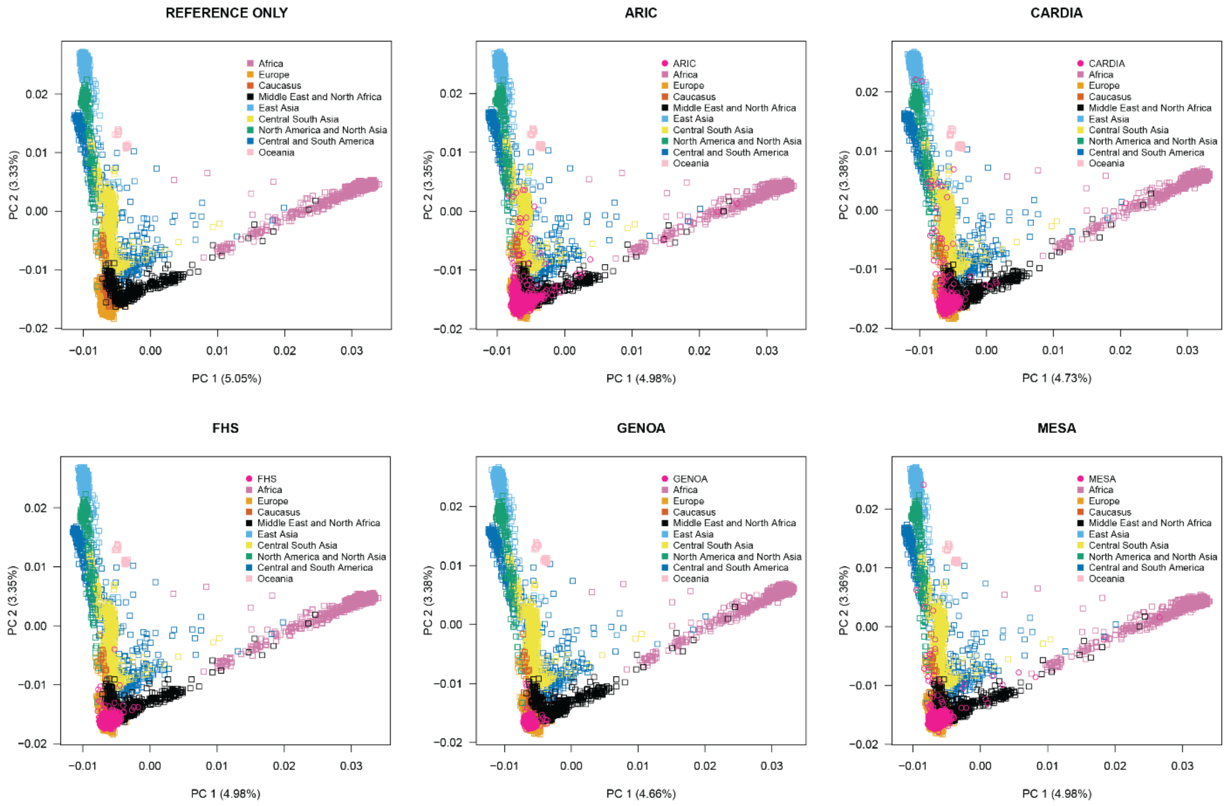

Fig. S2. Principal component analysis of the worldwide reference panel. Each European American cohort is projected onto the worldwide reference panel.

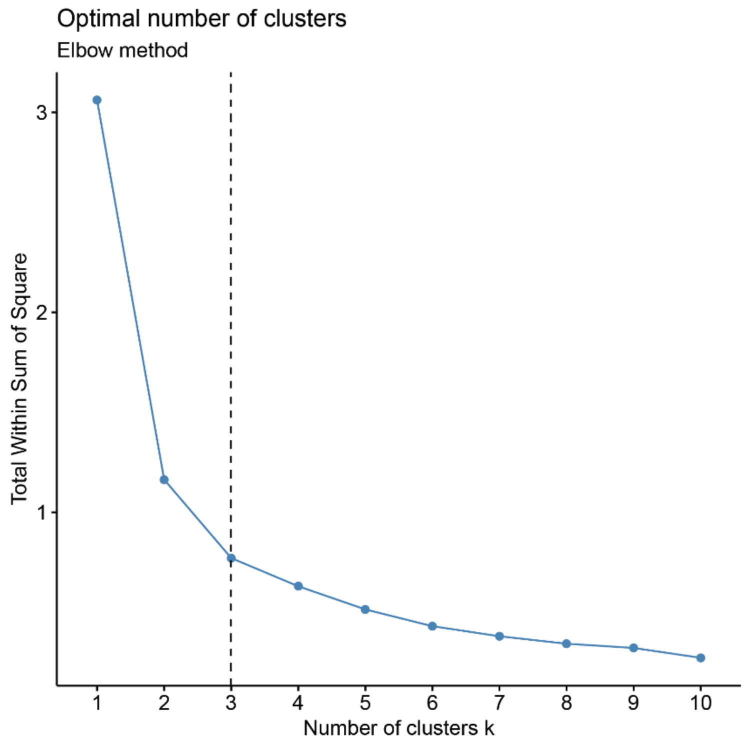

Fig. S3. Estimated number of clusters (k) using the elbow method. The number of clusters of European American individuals was estimated from the first two principal components derived from the projection of all European Americans onto the European reference panel.

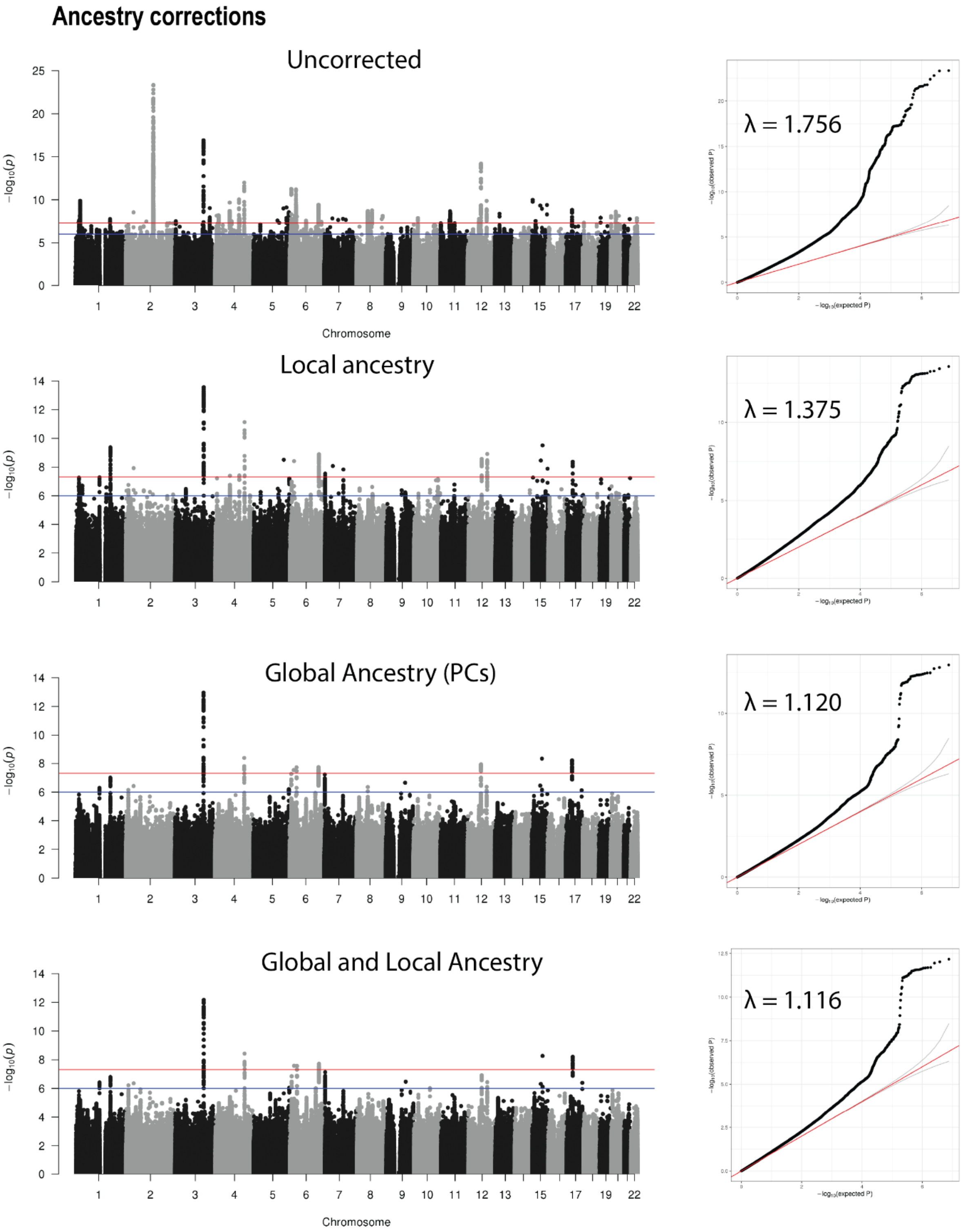

Fig. S4. Manhattan and q-q plots of GWAS of the trajectory of height accounting for different levels of control of population stratification. The models were adjusted for the top 12 Principal components significantly associated with height.

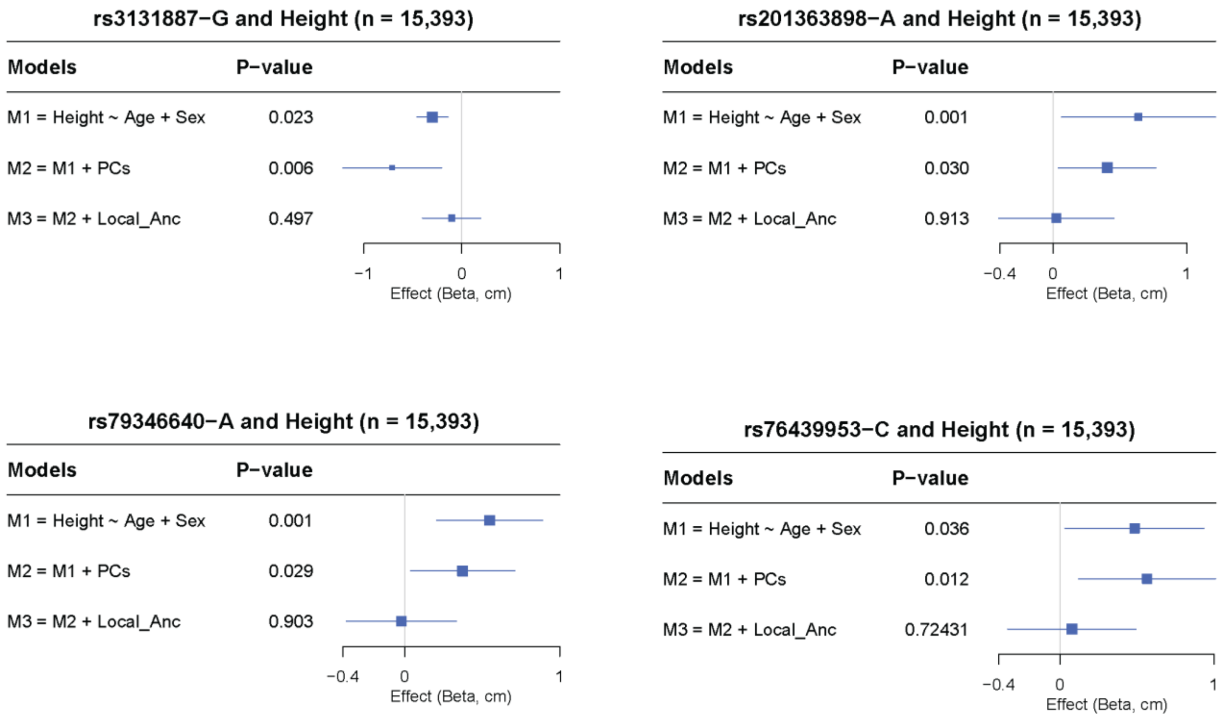

Fig. S5. Genetic loci with strong local ancestry effect in GWAS of height. The models were adjusted for the top 12 Principal components significantly associated with height. Forest plots show β values (95% confidence intervals) and *p*-values from linear models.
