# Supplementary material for: Unappreciated subcontinental admixture in Europeans and European Americans and implications for genetic epidemiology studies
Source: Nat Commun. 2023 Nov 7;14:6802. doi: 10.1038/s41467-023-42491-0 (PMC10630423; doi:10.1038/s41467-023-42491-0)
Supplement: Supplementary file 3 — Description of Additional Supplementary Files [file 41467_2023_42491_MOESM3_ESM.pdf]

Description of Additional Supplementary Files

File Name: Supplementary Data 1
Description: List of European populations and unrelated European samples included in our panel.

File Name: Supplementary Data 2
Description: Study characteristics for unrelated individuals in the European American cohorts.

File Name: Supplementary Data 3
Description: Genetic differentiation (F_{ST}) between world-wide populations.

File Name: Supplementary Data 4
Description: European ancestry estimates across the US European American cohorts.

File Name: Supplementary Data 5A
Description: Significant admixture f_3 statistics (Z score < -3) of the form f_3*(EUR_X , EUR_Y ; ARIC).*

File Name: Supplementary Data 5B
Description: Significant admixture f_3 statistics (Z score < -3) of the form f_3*(EUR_X , EUR_Y ; CARDIA).*

File Name: Supplementary Data 5C
Description: Significant admixture f_3 statistics (Z score < -3) of the form f_3*(EUR_X , EUR_Y ; FHS).*

File Name: Supplementary Data 5D
Description: Significant admixture f_3 statistics (Z score < -3) of the form f_3*(EUR_X , EUR_Y ; GENOA).*

File Name: Supplementary Data 5E
Description: Significant admixture f_3 statistics (Z score < -3) of the form f_3*(EUR_X , EUR_Y ; MESA).*

File Name: Supplementary Data 6
Description: Relatedness in the European American cohorts and European ancestry-assortative mating (F_{IT} *vs* F_{ST}).

File Name: Supplementary Data 7
Description: Admixture dating in our combined set of European Americans (Subgroup North) using 3-locus weighted LD decay.

File Name: Supplementary Data 8
Description: The association between rs4988235 and three biological traits (Height, LDL, and BMI) accounting for different levels of population stratification.

File Name: Supplementary Data 9
Description: Cohort-specific association analyses between height and rs4988235.

File Name: Supplementary Data 10
Description: Cohort-specific association analyses between LDL and rs4988235.

File Name: Supplementary Data 11
Description: Cohort-specific association analyses between BMI and rs4988235.

File Name: Supplementary Data 12
Description: Association analysis between height and genetic loci that were highly differenciated in Europe.

File Name: Supplementary Data 13
Description: Genetic loci with strong locus-specific ancestry effect in the GWAS of height.

File Name: Supplementary Data 14
Description: Mantel's correlation between genetic distance matrices derived from population-specific and the projection PCA approaches.
