# Supplementary material for: Unappreciated subcontinental admixture in Europeans and European Americans and implications for genetic epidemiology studies
Source: Nat Commun. 2023 Nov 7;14:6802. doi: 10.1038/s41467-023-42491-0 (PMC10630423; doi:10.1038/s41467-023-42491-0)
Supplement: Supplementary file 5 — Reporting Summary [file 41467_2023_42491_MOESM5_ESM.pdf]

nature portfolio

Corresponding author(s): Charles N. Rotimi

Last updated by author(s): Oct 11, 2023

Reporting Summary

Nature Portfolio wishes to improve the reproducibility of the work that we publish. This form provides structure for consistency and transparency in reporting. For further information on Nature Portfolio policies, see our Editorial Policies and the Editorial Policy Checklist.

Statistics

For all statistical analyses, confirm that the following items are present in the figure legend, table legend, main text, or Methods section.

n/a	Confirmed	
☐	☒	The exact sample size (*n*) for each experimental group/condition, given as a discrete number and unit of measurement
☐	☒	A statement on whether measurements were taken from distinct samples or whether the same sample was measured repeatedly
☐	☒	The statistical test(s) used AND whether they are one- or two-sided<br>*Only common tests should be described solely by name; describe more complex techniques in the Methods section.*
☐	☒	A description of all covariates tested
☐	☒	A description of any assumptions or corrections, such as tests of normality and adjustment for multiple comparisons
☐	☒	A full description of the statistical parameters including central tendency (e.g. means) or other basic estimates (e.g. regression coefficient) AND variation (e.g. standard deviation) or associated estimates of uncertainty (e.g. confidence intervals)
☐	☒	For null hypothesis testing, the test statistic (e.g. *F*, *t*, *r*) with confidence intervals, effect sizes, degrees of freedom and *P* value noted<br>*Give P values as exact values whenever suitable.*
☐	☐	For Bayesian analysis, information on the choice of priors and Markov chain Monte Carlo settings
☐	☒	For hierarchical and complex designs, identification of the appropriate level for tests and full reporting of outcomes
☐	☒	Estimates of effect sizes (e.g. Cohen's *d*, Pearson's *r*), indicating how they were calculated

Our web collection on statistics for biologists contains articles on many of the points above.

Software and code

Policy information about availability of computer code

Data collection	No software was used for data collection
Data analysis	PLINK 1.9, PLINK 2.0, ADMIXTURE 1.3, RFMix 1.5.4, ADMIXTOOLS 7.0.2, EAGLE 2.4, Minimac4, GENESIS 2.28.0, R 4.2.2, LaNeta, pipeline on GitHub (https://github.com/mateushg1/CRGGH/) for how to perform GWAS accounting for local ancestry, as well as how to perform ADMIXTURE and PCA projection analyses.

For manuscripts utilizing custom algorithms or software that are central to the research but not yet described in published literature, software must be made available to editors and reviewers. We strongly encourage code deposition in a community repository (e.g. GitHub). See the Nature Portfolio guidelines for submitting code & software for further information.

Data

Policy information about availability of data

All manuscripts must include a data availability statement. This statement should provide the following information, where applicable:

- Accession codes, unique identifiers, or web links for publicly available datasets
- A description of any restrictions on data availability
- For clinical datasets or third party data, please ensure that the statement adheres to our policy

The genome-wide and phenotypic data used in this manuscript are publicly available. Access to the European Americans from five genetic epidemiology cohorts was granted through dbGaP: ARIC (phs000090.v1.p1), CARDIA (phs000285.v3.p2), FHS (phs000007.v32.p13), GENOA (phs000379.v1.p1), and MESA (phs000209.v13.p3).

Publicly available data were retrieved from http://hgdownload.cse.ucsc.edu/gbdb/hg19/1000Genomes/phase3/, ftp://ngs.sanger.ac.uk/production/hgdp/hgdp_wgs.20190516/, https://reich.hms.harvard.edu/sites/reich.hms.harvard.edu/files/inline-files/EuropeFullyPublic.tar.gz, https://evolbio.ut.ee/caucasus/, and https://evolbio.ut.ee/jew/.

Research involving human participants, their data, or biological material

Policy information about studies with human participants or human data. See also policy information about sex, gender (identity/presentation), and sexual orientation and race, ethnicity and racism.

Reporting on sex and gender	Sex was self-identified and used as a covariate in a generalized linear mixed model. Gender was not used. The total number of males was 7,470 and the total number of females was 8,439.
Reporting on race, ethnicity, or other socially relevant groupings	We analyzed genome-wide data of 1,216 Europeans and 17,684 European Americans.
Population characteristics	The principal outcome was height, which is not a disease, so diagnosis and treatment are not applicable. Distributions of age, sex, height, body mass index, and low-density lipoprotein cholesterol for each study are provided in Supplementary Supplementary Data 2.
Recruitment	No new participants were recruited for this study.
Ethics oversight	US National Institutes of Health

Note that full information on the approval of the study protocol must also be provided in the manuscript.

Field-specific reporting

Please select the one below that is the best fit for your research. If you are not sure, read the appropriate sections before making your selection.

☒ Life sciences ☐ Behavioural & social sciences ☐ Ecological, evolutionary & environmental sciences

For a reference copy of the document with all sections, see nature.com/documents/nr-reporting-summary-flat.pdf

Life sciences study design

All studies must disclose on these points even when the disclosure is negative.

Sample size	No sample size calculation was performed. We analyzed genome-wide array and phenotypic data from 17,684 European Americans from five genetic epidemiology cohorts. The total sample size in meta-analysis was sufficient to reproduce previously described associations.
Data exclusions	All exclusion criteria were pre-established. Genotype data filtered by minor allele frequency of 1%, per genotype missingness 5%, per individual missingness 5%, and deviation from Hardy-Weinberg equilibrium less than 10^-6. Strand-ambiguous SNPs were removed. SNPs were pruned for strong linkage disequilibrium. Individuals were filtered for first and second degree relatives.
Replication	Association findings were previously described in the literature, so our study is a replication study, not a discovery study. Analysis was performed both by study and by meta-analysis of all studies combined. The workflow is reproducible. We tested different models with varying levels of ancestry adjustment (Suplementary Data 8-13) to show how the results can affected by population stratification.
Randomization	Participants were from observational studies and were not randomized. Covariates were included in association analyses using generalized linear mixed models.
Blinding	The study was not a clinical trial, so blinding was not relevant since there are no experimental treatments or interventions involved, and the samples are Non-personally identifiable, there is no need to blind participants or researchers.

Reporting for specific materials, systems and methods

We require information from authors about some types of materials, experimental systems and methods used in many studies. Here, indicate whether each material, system or method listed is relevant to your study. If you are not sure if a list item applies to your research, read the appropriate section before selecting a response.

Materials & experimental systems

n/a	Involved in the study
☒	☐ Antibodies
☒	☐ Eukaryotic cell lines
☒	☐ Palaeontology and archaeology
☒	☐ Animals and other organisms
☒	☐ Clinical data
☒	☐ Dual use research of concern
☒	☐ Plants

Methods

n/a	Involved in the study
☒	☐ ChIP-seq
☒	☐ Flow cytometry
☒	☐ MRI-based neuroimaging
